# Therapeutic strategies based on modified U1 snRNAs and chaperones for Sanfilippo C splicing mutations

**DOI:** 10.1186/s13023-014-0180-y

**Published:** 2014-12-10

**Authors:** Liliana Matos, Isaac Canals, Larbi Dridi, Yoo Choi, Maria João Prata, Peter Jordan, Lourdes R Desviat, Belén Pérez, Alexey V Pshezhetsky, Daniel Grinberg, Sandra Alves, Lluïsa Vilageliu

**Affiliations:** Department of Human Genetics, Research and Development Unit, INSA, Porto, Portugal; Department of Biology, Faculty of Sciences, Porto, Portugal; Departament de Genètica, Facultat de Biologia, Universitat de Barcelona, Barcelona, Spain; CIBER de Enfermedades Raras (CIBERER), Madrid, Spain; Institut de Biomedicina de la Universitat de Barcelona (IBUB), Barcelona, Spain; Department of Medical Genetics, Sainte-Justine University Hospital Centre, University of Montreal, Montreal, Canada; IPATIMUP, Porto, Portugal; Department of Human Genetics, Research and Development Unit, INSA, Lisbon, Portugal; Centro de Diagnóstico de Enfermedades Moleculares, Centro de Biología Molecular Severo Ochoa, UAM-CSIC, Universidad Autónoma de Madrid, Madrid, Spain; Department of Anatomy and Cell Biology, Faculty of Medicine, McGill University, Montreal, Canada

**Keywords:** Splicing mutations, Modified U1 snRNAs, Glucosamine, Sanfilippo C syndrome, Lysosomal storage disorder

## Abstract

**Background:**

Mutations affecting RNA splicing represent more than 20% of the mutant alleles in Sanfilippo syndrome type C, a rare lysosomal storage disorder that causes severe neurodegeneration. Many of these mutations are localized in the conserved donor or acceptor splice sites, while few are found in the nearby nucleotides.

**Methods:**

In this study we tested several therapeutic approaches specifically designed for different splicing mutations depending on how the mutations affect mRNA processing. For three mutations that affect the donor site (c.234 + 1G > A, c.633 + 1G > A and c.1542 + 4dupA), different modified U1 snRNAs recognizing the mutated donor sites, have been developed in an attempt to rescue the normal splicing process. For another mutation that affects an acceptor splice site (c.372-2A > G) and gives rise to a protein lacking four amino acids, a competitive inhibitor of the HGSNAT protein, glucosamine, was tested as a pharmacological chaperone to correct the aberrant folding and to restore the normal trafficking of the protein to the lysosome.

**Results:**

Partial correction of c.234 + 1G > A mutation was achieved with a modified U1 snRNA that completely matches the splice donor site suggesting that these molecules may have a therapeutic potential for some splicing mutations. Furthermore, the importance of the splice site sequence context is highlighted as a key factor in the success of this type of therapy. Additionally, glucosamine treatment resulted in an increase in the enzymatic activity, indicating a partial recovery of the correct folding.

**Conclusions:**

We have assayed two therapeutic strategies for different splicing mutations with promising results for the future applications.

**Electronic supplementary material:**

The online version of this article (doi:10.1186/s13023-014-0180-y) contains supplementary material, which is available to authorized users.

## Background

Sanfilippo syndrome or Mucopolysaccharidosis III (MPS III), is a group of autosomal recessive lysosomal storage disorders caused by mutations in genes encoding enzymes responsible for heparan sulfate degradation [1]. There are four types of the disease, depending on the gene affected. They all present similar clinical symptoms, including severe central nervous system degeneration accompanied by mild somatic manifestations [2]. Sanfilippo syndrome type C (MPS IIIC) is caused by mutations in the *HGSNAT* gene. This gene codes for the acetyl-CoA: α-glucosaminide N-acetyltransferase (EC 2.3.1.78), a protein localized in the lysosomal membrane which catalyses the acetylation of the terminal glucosamine residues of heparan sulphate prior to their hydrolysis by α-*N*-acetyl glucosaminidase [3]. The *HGSNAT* gene, identified by two independent groups in 2006 [4,5], is located at the chromosome 8 (8p11.1) and contains 18 exons. The cDNA encodes a polypeptide of either 635 or 663 amino acids, since there is a controversy concerning the real initiation codon [6,7]. To date, 64 mutations have been reported, 16 of which (25%) involve splicing alterations: 13 are described as splicing mutations and three as small deletions and duplications that affect the splicing process (HGMD®Professional Spring 2014.1 Release).

Splicing is an essential step for the expression of most of human genes, in which the 5’ and 3’ splice sites (ss), the branch point sequence and the polypyrimidine tract (both the last two within 50 nucleotides upstream of the 3’ ss) play a fundamental role. These sites present sequence variability throughout the human genome. The splicing process is conducted by the spliceosome, which is formed of five small nuclear RNAs (snRNAs) and more than 200 different proteins (reviewed in [8]). The U1 snRNA, which presents a partially complementary sequence to the 5’ ss, is essential for the recognition of the 5’ ss consensus motif (CAG/GTRAGT, exon/intron, R = purine). The U1 snRNP is composed of a 164-nt long U1 snRNA and several protein factors. The 5’ region of the U1 snRNA is involved in the recognition of the 5’ ss, with the C8 nucleotide being the one that binds the first nucleotide (G) of the intron (reviewed in [9]). Application of modified U1 snRNAs to improve recognition of mutated 5’ ss represents a new strategy for recovering the normal splicing process. They have been assayed as a therapeutic approach for different diseases and splicing mutations affecting different positions of the 5’ ss with variation in the efficacy of the treatment [10-19]. Recently, assays correcting an exogenous injected construct have been performed using modified U1 snRNAs in mice as a treatment for severe human factor VII deficiency [20].

In some cases, alternative splicing caused by specific mutations can give rise to misfolded proteins, which may be prone to rapid intracellular degradation (reviewed in [21]). Molecular chaperones are proteins that act on the correct folding of other polypeptides in cells. Pharmacological and chemical chaperones are small compounds that can be used, in a similar way, to avoid the misfolding of mutant proteins. They are principally potent enzyme inhibitors which interact specifically with their active sites to restore the correct folding and to increase stability [22]. In the case of lysosomal storage disorders, once in the lysosome, the enzyme substrate replaces the chaperone, thereby completing restoration of enzyme activity [23]. Aminosugars and iminosugars are the most common pharmacological chaperones used in enzyme enhancement therapy (EET) for lysosomal disorders. EET have been reported for several of these diseases including Fabry disease, G_M1_-gangliosidosis, Morquio B disease, Pompe disease, Gaucher disease, Krabbe disease, Niemann-Pick A/B and C diseases; as well as for many other types of disorders such as retinitis pigmentosa, cystic fibrosis, Parkinson’s disease, Alzheimer’s disease and cancer (reviewed in Ref. [21]). In the case of Sanfilippo syndrome type C, glucosamine, a competitive HGSNAT inhibitor, has been shown to increase residual enzyme activity in cultured skin fibroblasts from patients affected with a number of missense mutations [24].

In this work we focus on *HGSNAT* mutations that affect the splicing process. Three previously described splicing mutations [5,25,26] were the object of the study with different modified U1 snRNAs to improve recognition of the donor ss and enhance the correct splicing process. The studies were performed using cells transfected with minigene constructs bearing the specific mutation as well as cultured patients’ skin fibroblasts. Mutations include the most frequent change in Moroccan patients (c.234 + 1G > A), a mutation found in Spanish patients (c.633 + 1G > A) and a mutation found in a French patient (c.1542 + 4dupA). Furthermore, EET approach, was tested for a splicing mutation c.372-2A > G. This mutation is the most prevalent in Spanish and Portuguese patients [25,27] and affects the acceptor site at the end of the 4^th^ intron of the *HGSNAT* gene [27], thereby altering the splicing process. The use of a downstream alternative cryptic ss generates an mRNA with an in-frame deletion that codes for a protein with the loss of four amino acids (p. [L125_R128del]). Here, we show the effect of this mutation, which reduces enzyme activity, and the recovery of a part of that activity through treatment with glucosamine as a chaperone.

Our results show that, depending on the context of the mutated donor site, modified U1 snRNAs can be a promising therapeutic tool. The use of glucosamine as a chaperone improved enzyme activity, suggesting a therapeutic effect of this compound for Sanfilippo C patients.

## Methods

### Mutation analysis of the *HGSNAT* gene

This study included five MPS IIIC patients: three previously described, two Spanish and one Moroccan [25]; and two recently diagnosed, one French and one Portuguese, carrying mutations already reported by us [25,26] (Table 1). Studies were approved by the authors’ Institutional Ethics Committee and conducted under the Declaration of Helsinki. Patients were encoded to protect their confidentiality. Genetic analysis was performed using control and patients’ fibroblast cell lines as the source of RNA and genomic DNA whenever necessary. Total RNA was extracted using High Pure RNA Isolation Kit (Roche, Basel, Switzerland) and converted into cDNA using High-Capacity cDNA Reverse Transcription Kit (Life Technologies, Carlsbad, CA) following the manufacturers’ instructions. The RT-PCR amplifications were performed using the primers described in Additional file 1: Table S1 and information regarding specific conditions for each cDNA amplification and sequence analysis is available upon request.

**Table 1:** **Genotype and origin of MPS IIIC affected patients**

**Patient**	**Origin**	Allele 1*	Allele 2*	**Reference**
**SFCP**	Portugal	c.234 + 1G > A	c.234 + 1G > A	This study
**SFC3**	Morocco	c.234 + 1G > A	c.234 + 1G > A	[25]
**SFC6**	Spain	c.633 + 1G > A	c.1334T > C	[25]
**SFC7**	Spain	c.372-2A > G	c.372-2A > G	[25]
**SFC13**	France	c.1542 + 4dupA	c.1150C > T	This study

*Mutation nomenclature is based on cDNA sequence (NM_152419.2), with nucleotide +1 corresponding to the A of the ATG translation initiation codon.

### Minigene cloning and U1 snRNA expression constructs

For the *in vitro* splicing approaches, wild type (WT) and mutant minigenes were constructed for each mutation under study. A gene fragment including exon 2 and the flanking intronic regions was amplified from the DNA of the c.234 + 1G > A patient’s fibroblast cells using the primers described in Additional file 1: Table S1, and cloned into the TOPO vector (Life Technologies). The insert was excised with EcoRI, purified using Wizard® SV Gel and PCR clean-up system (Promega, Madison, WI), and subsequently cloned into the pSPL3 vector (Exon Trapping System, Life Technologies; kindly provided by Dr. B. Andresen) using the Rapid Ligation Kit (Roche Applied Science, Mannheim, Germany). Theclone containing the desired mutant insert in the correct orientation was identified by restriction enzyme analysis and DNA sequencing. The exon 6 (c.633 + 1G > A) and exon 15 (c.1542 + 4dupA), together with their intronic flanking sequences, were also cloned in the pSPL3 vector. These mutated minigenes were ordered from GenScript (Piscataway, NJ). In all cases, the WT minigenes were obtained by site-directed mutagenesis using the QuikChange II XL site-directed Kit (Agilent Technologies, Santa Clara, CA) following the manufacturer’s instructions and nucleotide changes were confirmed by sequencing analysis. The primers used are listed in Additional file 1: Table S1.

To express WT U1 snRNA (U1-WT), we used the vector pG3U1, which includes the sequence coding for human U1 ([28]; kindly provided by Dr. F. Pagani). The different U1 vectors adapted to the donor ss of *HGSNAT* exon 2, exon 6 and exon 15 were obtained by site-directed mutagenesis using the QuikChange II XL site-directed Kit (Agilent Technologies). For each construct (U1 suppressor 1 to 9) the PCR reaction was performed with the specific primers shown in Additional file 1: Table S1. The desired mutations were confirmed by sequence analysis.

### Cell culture and U1 transfection experiments

To perform the splicing assays, COS-7 cells and fibroblasts were grown in Dulbecco’s modified Eagle’s medium (Sigma-Aldrich, St. Louis, MO) supplemented with 10% fetal bovine serum (Life Technologies) and 1% PenStrep (Life Technologies) at 37°C with 5% CO_2_. For co-transfection experiments, COS-7 cells at 90% of confluence were transfected in 6 or 12-well plates with either 1 or 2 μg of each WT or mutant minigene and 1 to 4 μg of each different mutation adapted U1 snRNA vector, using Lipofectamine 2000 (Life Technologies) according to the manufacturer’s protocol. When required, the amount of DNA was adjusted with the pSPL3 empty vector. For splicing analysis of the endogenous *HGSNAT* transcripts, healthy control and patients’ fibroblasts at 90% of confluence were transfected in 6-well plates with 1, 2.5 or 3.5 μg of each modified U1 snRNA vector using either Lipofectamine 2000 or LTX (Life Technologies) according to the manufacturer’s instructions. To estimate transfection efficiency, healthy control and patients’ cells were transfected with a control plasmid encoding either GFP or RFP and fluorescent cells were monitored by microscopy.

### RT-PCR transcript analysis after transfection of modified U1 snRNAs

Cells were harvested 24 or 48 h after transfection. Total RNA was extracted using High Pure RNA Isolation Kit (Roche Applied Science) and converted into cDNA using High-Capacity cDNA Reverse Transcription Kit (Life Technologies). The RT-PCR splicing analysis for minigene transfections was performed using the typical pSPL3 primers SD6 and SA2 (Additional file 1: Table S1). For the endogenous experiments, RT-PCRs were performed using the following primers: HGSNAT-Exon 2F/HGSNAT-Exon 3R for mutation c.234 + 1G > A, HGSNAT-Exon 5F/HGSNAT-Exon 13R for c.633 + 1G >A/c.1334T > C and HGSNAT-Exon 12F/HGSNAT-Exon 16R for the c.1542 + 4dupA/c.1150C > T mutation. In the case of the c.234 + 1G > A mutation, the forward primer was designed to anneal in the middle of exon 2 to amplify only the transcripts in which the correct splicing process was recovered. For the other mutations, the last nucleotide of one of the primers corresponded to the point mutation of the other allele (but with the WT nucleotide) to favour that only the cDNA from the splice-mutation allele was amplified. The RT-PCR products were sequenced to confirm their identity. All the primers used are listed in Additional file 1: Table S1 and information regarding the amplification conditions is available upon request.

### Expression of recombinant human mutant and wild type *HGSNAT* in COS-7 cells

COS-7 cells cultured in Eagle’s minimal essential medium supplemented with 10% fetal bovine serum to 70% confluence were transfected with the pcTAP-HGSNAT plasmid and mutant pcDNA-HGSNAT-L125_R128del plasmid as previously described [24]. 48 h after transfection the cells were harvested and analysed for N-acetyltransferase enzymatic activity or by Western blot.

### Glucosamine-mediated refolding of mutant HGSNAT

Twenty-four h after transfection with pcTAP-HGSNAT or mutant pcDNA-HGSNAT-L125_R128del plasmids, COS-7 cells were grown for 72 h in Eagle’s minimal essential medium supplemented with 10% fetal bovine serum containing 10 mM glucosamine in 6-well plates. Then the cells were kept for another 24 h in the normal culture medium without glucosamine. The cells were harvested, lysed by freeze-thaw in 750 μl of water and assayed for HGSNAT activity. Three independent experiments (each with 2 cell plates) were performed on 3 different occasions.

Confluent primary cultures of skin fibroblasts of the MPS IIIC patient homozygous for the c.372-2A > G mutation and healthy control fibroblasts (n = 5) were grown in 6-well plates for 72 h in Dulbecco’s minimal essential medium supplemented with 10% fetal bovine serum containing 10 mM glucosamine. Then the cells were kept for another 24 h in the medium without glucosamine, harvested, lysed in 500 μl of water and assayed for HGSNAT activity. Three independent experiments (each with 2 cell plates) were performed on 3 different occasions.

### Enzyme assay

HGSNAT N-acetyltransferase enzymatic activity was measured using the fluorogenic substrate 4-methylumbelliferyl β-D-glucosaminide (4MU-β-GlcN; Moscerdam, Oegstgeest, Netherlands) as previously described [24]. The protein concentration was measured according to the method of Bradford using a commercially available reagent (BioRad, Hercules, CA).

### Statistical analysis

Statistical analysis of the data has been performed by two-tailed unpaired t-test using the Prism GraphPad software.

### Western blotting

Cell lysates from 3 independent transfections (20 μg of total protein each) were analysed by Western blot as previously described [24] using rabbit polyclonal antibodies raised against human HGSNAT Q52-N156 peptide (Sigma-Aldrich HPA029578, dilution 1:5000, incubation overnight at 4°C). Detection was performed with an anti-rabbit IgG antibodies-HRP conjugate (ref. 7074S, Cell Signalling, Beverly, MA), and the enhanced chemiluminescence reagent (ref. 32106, Thermo Scientific, Waltham, MA).

## Results

### Mutation analysis in MPS IIIC patients’ fibroblasts

**Figure 1:**
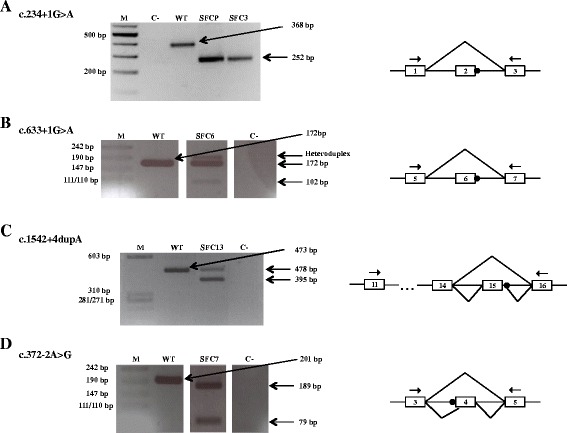
**Analysis of alternative** ***HGSNAT*** **transcripts from the mRNA of five MPS IIIC patients by RT**-**PCR.** RT-PCR amplification of mutations c.234 + 1G > A **(A)**, c.633 + 1G > A **(B)**, c.1542 + 4dupA **(C)**, and c.372-2A > G **(D)**, and of the WT allele, using the primers indicated in the schemes by arrows. The different splicing patterns are depicted in the schemes (right), in which the mutations are indicated by black circles. M: marker, C-: negative control, SFCP, −3, − 6, −13, −7: patients. Boxes in the schemes correspond to exons.

This study involved four splicing mutations found in five different patients: SFCP and SFC3 (both homozygous for c.234 + 1G > A), SFC6 (compound heterozygous for c.633 + 1G > A and the missense mutation c.1334T > C; p.L445P), SFC13 (compound heterozygous for c.1542 + 4dupA and the nonsense mutation c.1150C > T; p.R384*) and SFC7 (homozygous for c.372-2A > G) (see Table 1). For patients SFC3 (previously reported [25]) and SFCP (novel patient), the RT-PCR analysis of cDNA with primers in exon 1 and 3 revealed a fragment skipping exon 2 (252 bp) due to the presence of the c.234 + 1G > A mutation in homozygosity (Figure 1A). For patient SFC6 (previously described [25]) the cDNA analysis with primers in exons 5 and 7 showed two transcripts: one of a normal length of 172 bp arising from the missense mutation allele (c.1334T > C; p.L445P); and a second one resulting from the skipping of exon 6 (102 bp), due to the splicing alteration c.633 + 1G > A in the other allele (Figure 1B). For a novel patient, SFC13, bearing the intron 15 mutation c.1542 + 4dupA in compound heterozygosity with the c.1150C > T nonsense change in exon 11, the RT-PCR analysis with primers in exons 11 and 16 revealed two aberrant transcripts. The first one showed the skipping of exon 15 (395 bp), and in the second, exon 15 was extended for the first 5 nucleotides of intron 15 (478 bp) due to the presence of a cryptic ss in the beginning of this intron (Figure 1C). For the fifth patient, SFC7 (previously reported [25]), who is homozygous for the c.372-2A > G mutation, the transcript analysis using primers in exons 3 and 5 revealed the presence of two transcripts: one of lower size (79 bp) in which exon 4 was skipped; and the other of higher size (189 bp) corresponding to a fragment with the deletion of the first 12 nucleotides of exon 4 due to the use of a cryptic 3’ ss localized downstream of the constitutive one (Figure 1D), which gives rise to the p.[L125_R128del] mutant protein.

### Development of splicing therapy approaches for *HGSNAT* gene mutations: minigene assays with modified U1 snRNA vectors

**Figure 2:**
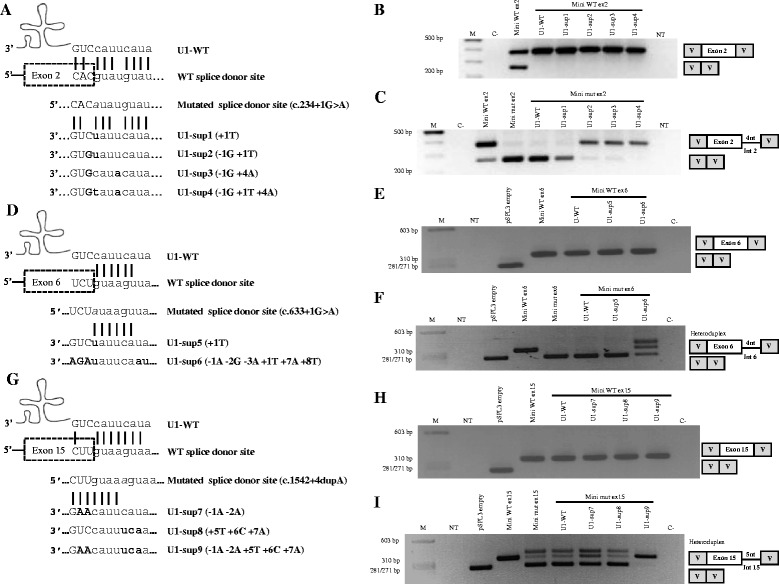
**Minigene therapeutic approaches with U1 adaptations to correct the pathogenic effect of different donor ss mutations on the** ***HGSNAT*** **gene. (A)**, **(D)** and **(G)** Schematic illustrations of base pairing between the wild type U1 (U1-WT) and the 5’ ss of wild type and mutant exons 2, 6 and 15 of the *HGSNAT* gene. The position of each mutation in the 5’ ss is marked in grey and it is in italics. The different U1 snRNAs used for each mutated 5’ ss of *HGSNAT* (designated as U1-sup, for suppressor) are also shown. The U1 sequence changes are illustrated in bold. **(B)**, **(C)**, **(E)**, **(F)**, **(H)** and **(I)** RT-PCR analysis of COS-7 cells in which splicing reporter minigenes for each mutation were co-transfected with the different modified U1 constructs as appropriate. Neither the U1-WT nor the various adapted U1s showed an effect on the splicing of exon 2, 6 or 15 wild type minigenes **(B, E and H)**. In the case of the mutant minigenes, some of the U1 co-transfections [U1-sup2, 3 and 4 **(C)**; U1-sup6 **(F)**; U1-sup7, 8 and 9 **(I)**] changed the splicing pattern, not to the correct one, but mainly towards an alternative pattern using a “gt” 5’ ss immediately downstream of the constitutive one. Importantly, the wild type minigene for exon 2 (Mini WT ex2) shows an extra lower band due to the amount of empty vector added to adjust the total quantity of DNA. Sequencing results for all RT-PCR products are illustrated by schematic drawings. M: molecular weight marker; NT: non-treated cells; C-: negative control.

To reproduce the splicing defects in a cellular model that could be used to test U1 snRNA overexpression as a therapeutic strategy we constructed several mutant minigenes in the pSPL3 vector and expressed them in COS-7 cells. Each of the minigenes bore either one of the three mutations that affect the donor ss or the WT allele. The post-transfection cDNA analysis and sequencing revealed the skipping of exon 2 and exon 6 in the cases of the c.234 + 1G > A and c.633 + 1G > A mutant minigenes, respectively (Figure 2C,F). For the c.1542 + 4dupA minigene, the cDNA showed a band that corresponded to the skipping of exon 15 and another band that corresponded to the inclusion of this exon plus the first five nucleotides of intron 15 (Figure 2I). Meanwhile, the constructs with the WT sequences (relative to each mutation) revealed a single transcript for each of the three cases with the inclusion of exon 2, exon 6 or exon 15, respectively. These results show that the minigene-derived splicing patterns closely resemble the patterns observed in control and patients’ cDNAs obtained from fibroblasts. Thus, the minigenes are reliable tools to test and optimize the overexpression of modified U1 snRNAs in our attempts to correct the splicing defect.

To evaluate the efficiency of different U1s for correcting missplicing of the *HGSNAT* exons 2, 6 and 15, several constructs, with different degrees of complementarity to each mutated donor ss, were generated (Figure 2A,D,G). For each specific case, the WT and mutant splicing reporter constructs were co-transfected with the different U1 snRNAs into COS-7 cells for 24 and 48 h. RT-PCR analysis was then performed and showed that U1-WT and the different U1 snRNA modifications do not interfere with the splicing pattern of any WT minigene (Figure 2B,E,H).

In the case of the mutant minigenes, different splicing patterns were observed after overexpression of the different U1 snRNAs. In the case of the mutant c.234 + 1G > A minigene, the cDNA analysis revealed no change in splicing after co-expression with the U1-WT and U1-sup1 (+ 1T) constructs. With the remaining modified U1s [U1-sup2 (−1G + 1T); U1-sup3 (−1G + 4A); U1-sup4 (−1G + 1T + 4A)], a band of the size of 376 bp, expected for the normal splicing, was observed. However, sequence analysis showed that the fragment included exon 2 and the first four base pairs of intron 2 (ATAT), due to the use of an alternative donor site with the canonical “gt” in positions +5 and +6. A very faint band corresponding to exon 2 skipping was still observed when U1-sup2 and U1-sup3 were overexpressed (Figure 2C).

For the mutant c.633 + 1G > A minigene, the splicing pattern was not altered after overexpression of U1-WT and U1-sup5 (+ 1T), while an apparently normal band was detected with the U1 matching all the nucleotides of the mutated donor ss [U1-sup6 (- 1A - 2G - 3A + 1T + 7A + 8T)]. Sequencing of this band demonstrated aberrant splicing which includes, apart from exon 6, the first four nucleotides (ATAA) of intron 6. Again, this was due to the presence of an alternative donor site with the canonical “gt” in positions +5 and +6. A band of low molecular weight, which corresponded to the skipping of exon 6 was also detected upon U1-sup6 treatment (Figure 2F).

In the c.1542 + 4dupA mutant minigene, the co-transfection of the totally complementary U1 [U1-sup9 (- 1A - 2A + 5T + 6C + 7A)] induced the appearance of a single band of the predicted normal size. Sequence analysis revealed an abnormal fragment including exon 15 and the first five base pairs (GTAAA) of intron 15. As in the two previous cases, this was due to the presence of a “gt” in positions +6 and +7 (the duplication of an A in this mutant allele moved the “gt” from the original +5 and +6 positions). This band was also observed in the untreated mutant minigene. The overexpression of U1-sup7 (−1A-2A) produced an increase in intensity of the band that corresponded to the use of the alternative site; while no effect of U1-sup8 (+ 5T + 6C + 7A) or U1-WT was detected (Figure 2I).

### Development of splicing therapy approaches for *HGSNAT* gene mutations: treatment of patients’ cells with modified U1 snRNA vectors

**Figure 3:**
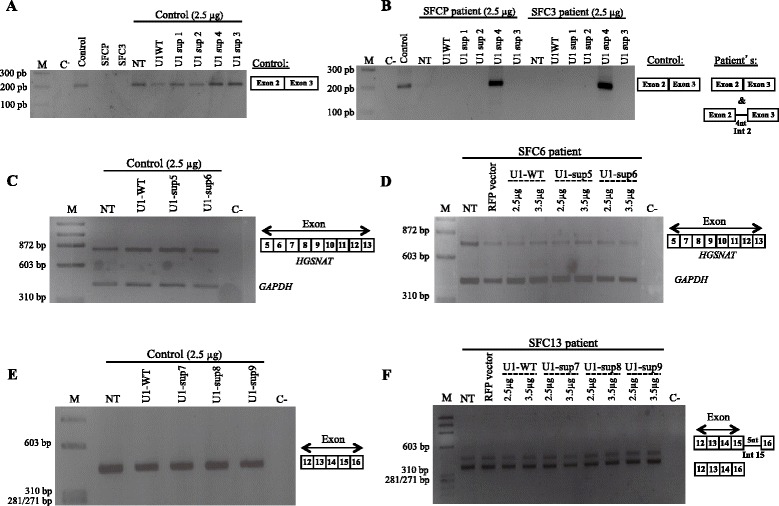
**Analysis by RT-** **PCR of the endogenous splicing pattern of control and patients SFCP**, **SFC3**, **SFC6 and SFC13 derived fibroblasts after transfection with different U1 isoforms. (A)**, **(C)** and **(E)** The constitutive splicing of exons 2, 6 and 15 of the *HGSNAT* gene was not altered in control fibroblasts after overexpression of U1-WT or any of the modified U1 constructs. **(B)** In the patients SFCP and SFC3, bearing the homozygous mutation c.234 + 1G > A, only the fully adapted U1 (2.5 μg of U1-sup4) resulted in partial correction of exon 2 skipping. The same result was obtained with 1 or 3.5 μg of the U1-sup4 construct (data not shown). **(D)** and **(F)** For patients SFC6 and SFC13, bearing genotypes c.633 + 1G >A/c.1334T > C and c.1542 + 4dupA/c.1150C > T, respectively, the transfection of 2.5 or 3.5 μg of U1-WT or any generated U1 suppressor vector did not produced any change in the endogenous aberrant splicing pattern. Sequencing results for all RT-PCR products are illustrated by schematic drawings. M: molecular weight marker; NT: non-treated cells; C-: negative control; RFP: red fluorescent protein.

Despite the data of minigene assays showing that the three ss defects were not corrected by the expression of the different modified U1 vectors, we explored the feasibility of this approach to correct the endogenously misspliced *HGSNAT* transcripts through transfection of the same U1 variants in patients’ derived cell lines. The spliced transcripts obtained after transfection of each fibroblast cell line were analysed by RT-PCR (Figure 3A-F).

Interestingly, for patients SFCP and SFC3, homozygous for the c.234 + 1G > A mutation, treatment with different quantities of fully adapted U1 [U1-sup4 (−1G + 1T + 4A)] resulted in a partial recovery from the splicing defect. Sequence analysis revealed two different sequences: one with the normal splicing; and the other which included the first four base pairs of intron 2 (Figure 3B and Additional file 2: Figure S1), as detected in the minigene approaches in COS-7 cells (Figure 2C). To estimate the levels of correct splicing, the obtained PCR product was cloned in a pUC19 vector and amounts of around 50% were detected for the correct sequence (10 out of 22 clones with the correct spliced fragment). Due to this partial correction obtained, an experiment to measure the enzymatic activity in patient’s cells after U1-sup4 transfection was carried out. However, no improvement in enzyme activity was observed. The values obtained for both patients, without treatment and using 2.5 μg U1sup4, were negligible and far below the normal range of controls (1.95-13.4 nmol/17 h/mg). The expression of the remaining U1 modifications had no detectable effect on exon 2 splicing.

In the case of the patients SFC6 (c.633 + 1G > A/c.1334T > C) and SFC13 (c.1542 + 4dupA/c.1150C > T), the RT-PCR analysis showed no effect of any modified U1 snRNA on the endogenous splicing process (Figure 3D,F). Similarly, WT *HGSNAT* gene splicing was not affected by any of the U1 isoforms (Figure 3A,C,E).

To rule out the possibility of the negative results being due to poor fibroblast transfection efficiency, fluorescence expression GFP/RFP vectors were used; efficient acquisition of the vectors was observed (data not shown). The uptake of the U1 snRNA was confirmed by PCR (Additional file 3: Figure S2). Moreover, the electroporation technique was also tried but no improvement in transfection efficiency was achieved (data not shown).

### Analysis of the protein encoded by the mutant *HGSNAT* cDNA with 12 nucleotides deleted

**Figure 4:**
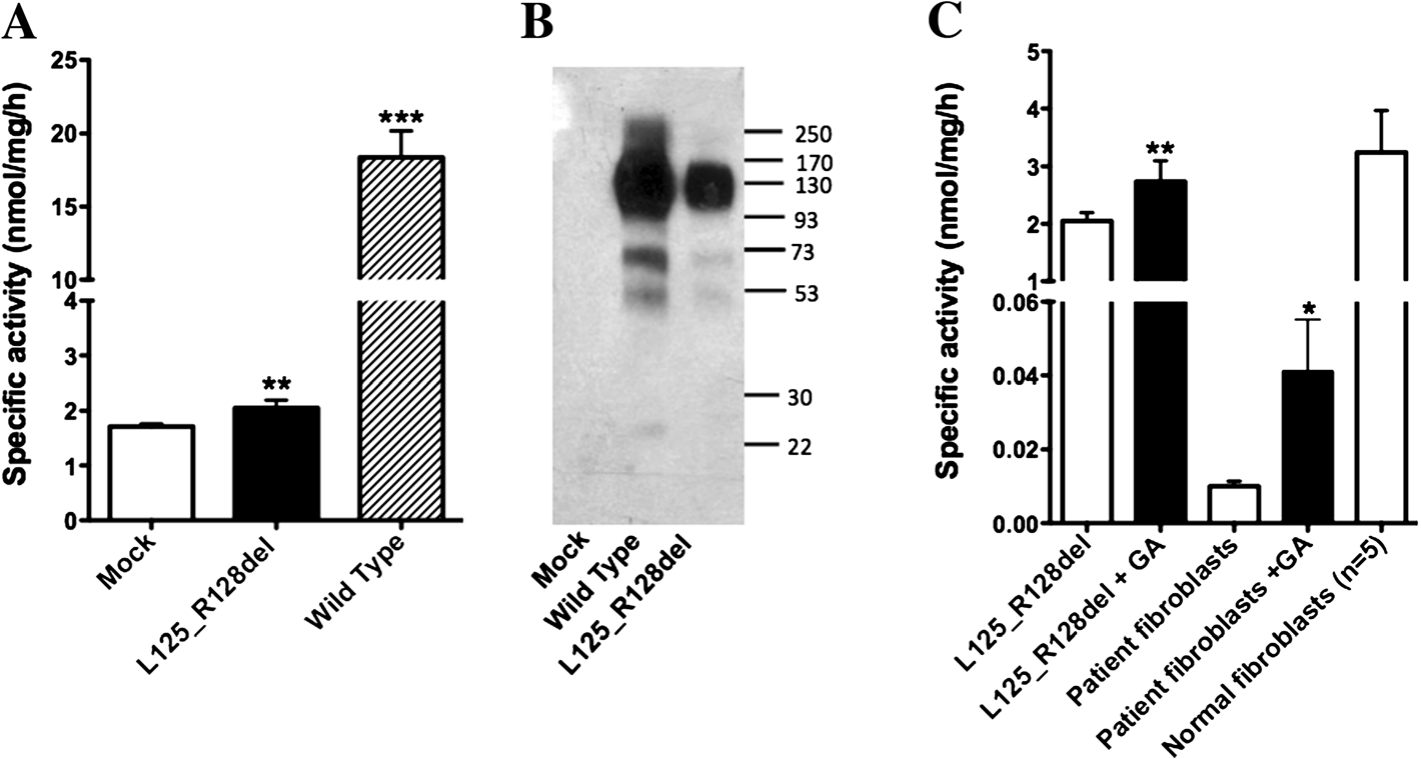
**Enzymatic activity of mutant HGSNAT**-**L125**_**R128del protein can be partially restored by the pharmacological chaperone,** **glucosamine. (A)** N-acetyltransferase activity of COS-7 cells transfected with the pcDNA-HGSNAT-L125_R128del plasmid is significantly increased compared to that of cells transfected with the empty pcDNA plasmid (mock). The data show means (±S.D.) of individual measurements. Three transfections (each in duplicate) were performed on separate occasions.** and ***: statistically different from mock-transfected cells (p < 0.01 and p < 0.001, respectively) according to unpaired t-test. **(B)** COS-7 cells transfected with the pcDNA-HGSNAT-L125_R128del plasmid produce 160 kDa dimmers and 78 kDa monomers of HGSNAT precursor protein but show drastically reduced amounts of 44 kDa and 25 kDa mature HGSNAT chains produced by intra-lysosomal enzymatic cleavage. Panel shows representative data of 3 independent transfections. **(C)** N-acetyltransferase activity of COS-7 cells transfected with the pcDNA-HGSNAT-L125_R128del plasmid and of cultured primary fibroblasts of the patient homozygous for the c.372-2A > G mutation is significantly increased after treating the cells in culture with 10 mM glucosamine for 72 h (+GA). The data show means (±S.D.) of individual measurements. Three independent experiments measurements were performed each of them with 2 cell plates. * and **: statistically different from untreated cells (p < 0.05 and p < 0.01, respectively) by unpaired t-test.

In order to test whether mutant cDNA with a 12-nucleotide in-frame deletion (encoding p.[L125_R128del]) produces a stable protein and, if so, whether it is enzymatically active, we transfected cultured COS-7 cells with a corresponding pcDNA-HGSNAT-L125_R128del plasmid and measured N-acetyltransferase activity in the cell homogenate. Cells transfected with an empty plasmid or those transfected with the pcTAP-HGSNAT plasmid described previously [6] encoding WT human HGSNAT were used as positive and negative controls, respectively. Our data (Figure 4A) showed a small but significant increase of N-acetyltransferase activity in the cells transfected with the mutant *HGSNAT* compared to that in the cells transfected with the empty plasmid. The activity in the cells transfected with WT pcTAP-HGSNAT increased about 10-fold (Figure 4A).

We further analysed cell homogenates by Western blot and detected a cross-reacting band in the cells transfected with both WT and mutant plasmids (Figure 4B). The amount of 160 kDa dimer of the HGSNAT precursor was comparable in both homogenates, but the amount of 44 kDa and 25 kDa enzymatically cleaved mature HGSNAT chains was reduced in the cells transfected with the pcDNA-HGSNAT-L125_R128del plasmid. Since the enzymatic cleavage of the HGSNAT precursor into the 2-chain form occurs in the lysosome [6], this result is consistent with misfolding of the majority of the HGSNAT-L125_R128del mutant and its retention in the endoplasmic reticulum (ER), as previously demonstrated for most HGSNAT mutants with amino acid substitutions [24].

### Partial recovery of the enzyme activity of p.[L125_R128del] by glucosamine as a pharmacological chaperone

Our previous data demonstrated that the treatment of cultured cells of MPS IIIC patients bearing missense mutations with the competitive HGSNAT inhibitor (Ki = 0.28 mM) glucosamine, significantly increased the level of the residual N-acetyltransferase activity [24]. In order to determine whether glucosamine can also restore the folding and activity of the p.[L125_R128del] mutant protein, we treated COS-7 cells transfected with the pcDNA-HGSNAT-L125_R128del plasmid and cultured primary fibroblasts of the patient homozygous for the c.372-2A > G mutation with 10 mM glucosamine. Our data (Figure 4C) show that glucosamine significantly increases residual N-acetyltransferase activity in both COS-7 cells transfected with the pcDNA-HGSNAT-L125_R128del plasmid and in the patient’s fibroblasts. This suggests that the active conformation of the mutant HGSNAT can be stabilized by glucosamine, resulting in part of the mutant enzyme pool being properly processed and targeted at the lysosomes.

## Discussion

In this study we describe two therapeutic approaches specific for several splicing mutations in the *HGSNAT* gene that lead to defects in mRNA processing in five Sanfilippo C patients, each carrying at least one splicing mutation. To our knowledge, this is the first attempt to treat Sanfilippo syndrome type C splicing mutations with modified U1 snRNAs. The chaperone treatment, the second therapeutic strategy examined in the present work, was previously tested *in vitro* for several missense Sanfilippo C syndrome mutations with promising results [24] and it is applied here for the treatment of a mutant protein lacking four amino acids.

For patients carrying mutations in the donor ss (SFCP, SFC3, SFC6 and SFC13), modified U1 snRNAs have been tested as a therapeutic tool to recover the normal splicing process. This approach has previously been tested for different mutations in several disorders showing different efficiencies at rescuing the normal transcripts [10-19]. A total of 9 different U1s have been developed, as well as the U1-WT. None of them affected the normal splicing process in WT minigenes or healthy control fibroblasts when overexpressed.

Few assays to correct a +1 5’ ss mutation have been reported. In some of those, the efficiency of modified U1 snRNAs for splicing mutations in canonical positions +1 and +2 has been shown to be inexistent [10,13]. However, Hartmann *et al*. [19] showed a partial correction of a +1 mutation in a case in which some degree of normal splicing was conserved in the mutant allele and the alternative donor site with a “gt” dinucleotide in positions +5 and +6 presented a low score according to different predictors. In this report, we present three cases with splicing mutations at position +1, two homozygous patients with the c.234 + 1G > A mutation (SFCP and SFC3) and one heterozygous patient with the c.633 + 1G > A mutation (SFC6). When we analysed the treatment efficiency of different modified U1 snRNAs in minigene constructions carrying these mutations, we were not able to detect restoration of the normal splicing process. Instead, we detected alternative splicing patterns due to the use of highly conserved “gt” nucleotides at positions +5 and +6 as a donor site, giving rise to a transcript that includes 4 intronic nucleotides. This result was obtained using three of the four modified U1 snRNAs for the c.234 + 1G > A mutation and the total complementary U1 snRNA for the c.633 + 1G > A mutation. Due to the presence of these “gt” dinucleotides at position +5 and +6 in many introns, it is important to sequence putative rescued transcripts to check whether this alternative site was used.

Despite these results, and taking into account that minigenes only included partial intronic sequences which could lack some splicing regulatory sites and that they were assayed in non-human cells, modified U1 snRNAs were tested on patients’ fibroblasts. Partial rescue (almost 50%) of the normal splicing for the c.234 + 1G > A mutation was observed in patients SFCP and SFC3 overexpressing the U1-sup4. In the case of the c.633 + 1G > A mutation (patient SFC6), no rescue was observed after the modified U1 snRNAs overexpression. Analysis of the ss sequences using the Human Splicing Finder predictor [29], indicated that the alternative site of patient SFC6 had a high score (68.17/100), while that of patients SFCP and SFC3 had a null score. This could explain the difference in the rescue results between these two “+1” mutations: the mutant allele of patient SFC6 would efficiently use the alternative site and, thus, would not be rescued.

The molecular reason why the modified U1 promotes, in general, a new splicing process using the +5 + 6 “gt” donor site when its sequence perfectly matches the mutated +1 + 2 site remains unclear. One explanation could be the involvement of U6 snRNA complementarity, in addition to that of U1, in the choice of alternative ss in proximity to the normal one, which has been previously described [30,31].

The rescue of the splicing mutation shown here is one of the few positive results for a “+1” mutation using a modified U1 snRNA and the first one performed on an allele that did not produce any of the correctly spliced mRNAs when untreated. It is important to note that a few natural U2-type 5’ ss that present “au” instead of the normal “gu” at the first two nucleotides of the intron have been described [32,33]. These introns present “ac” instead of “ag” at the last two nucleotides. Thus, one could think that mutated “au” sites could promote an alternative splicing using an “ac” acceptor site. However, for this +1 mutation the “au-ac” alternative splicing type was not observed. On the contrary, we found that the “au” 5’ ss correctly paired with the normal “ag” and not with a possible cryptic “ac” 3’ ss, restoring the normal splicing of intron 2.

Further studies are needed to improve the efficacy and to test the toxicity and side effects of U1 overexpression in order to confirm the feasibility of the use of this modified U1 snRNAs in therapy. Overexpression of U1 snRNA vectors introduced with adeno-associated virus into mice liver has been shown to be toxic at high viral doses but safe at low doses, suggesting the viability of this treatment when low amounts of U1 snRNA viral particles are injected [20].

In the case of the c.1542 + 4dupA mutation (patient SFC13), it is important to note that the canonical “gt” site is not affected by the mutation. However, in this case there is also another “gt” dinucleotide in positions +6 and +7 (one nucleotide downstream due to the duplication). The minigene analysis showed that the splicing that uses the alternative donor site occurs, even without treatment, together with that causing exon 15 skipping. This alternative splicing is slightly enhanced with U1-sup7 and greatly enhanced with U1-sup9. Many different ss predictors were used in order to estimate the score for each donor site in this case (Additional file 4: Table S2). Clearly, in the absence of mutation, the scores of both sites are similar; but when the mutation is present, the normal site presents a lower score while the alternative site increases its score. This is consistent with the fact that in the presence of the mutation the alternative “gt” site is the only one used (see Figure 2I). As it was previously discussed, it remains unclear why the modified U1 snRNAs, designed to perfectly match the mutated site, enhance the splicing using the alternative site. When the modified U1 snRNAs were tested on patient’s fibroblasts, no rescue was detected: either for the normal mRNA or for the one including the five intronic nucleotides. The latter was detected without any treatment, indicating that it takes place in normal conditions in the patient’s cells. In any case, the alternative transcript would not restore the enzyme activity since it does not keep the frame, so it could not have any therapeutic use. Many different mutations in the +3 to +6 positions have been partially or totally rescued using U1 snRNAs [10-13,15,17,18], while one +5 mutation was not corrected [15]. Our current results point to the importance of the presence of an additional “gt” dinucleotide in the region which, depending on the sequence context, may be used as an alternative donor site. In previous reports where mutations have been partially or totally corrected and the “gt” dinucleotide was present at positions +5 and +6 [15,19], the alternative site presented a low score in accordance with different predictors, in contrast to the cases described here.

Finally, we attempted to rescue the c.372-2G > A mutation (present in SFC7), using molecular chaperone. This mutation gives rise to two different splicing processes in fibroblasts: one causing exon 4 skipping; the other using an alternative acceptor site, 12 nucleotides downstream of the normal site and producing a transcript with an in-frame deletion. We showed that this alternative splicing produces a protein lacking 4 amino acids p. [L125_R128del] that has some residual activity but the majority of the protein does not reach the lysosome, remaining in the ER due to its misfolding.

In order to restore the correct protein folding, cells transfected with the plasmid carrying the mutant protein and the patient’s fibroblasts were treated with glucosamine chaperone, which resulted in a significant increase in the residual HGSNAT activity. This indicates the feasibility of this therapeutic approach for patients carrying this splicing mutation. Different chaperones assays for lysosomal disorders have been performed before and some have been tested in humans, showing their safety and potential as a therapeutic tool (reviewed in Ref. [21]).

## Conclusions

In conclusion, the results of two therapeutic approaches for different splicing mutations varied depending on the nature of the mutation. For the treatment of donor ss mutations, U1 snRNAs could represent a feasible option. This would depend on the presence of alternative donor sites close to the normal site that could interfere with the correction process and, thus, with the success of the therapy. This is important since many introns present a “gt” in positions +5 and +6. In the present study, we have shown that it is possible to partially recover the normal splicing process for +1 mutations, which was reported only once before. Additionally, a chaperone treatment using glucosamine for a mutant protein with a loss of 4 amino acids, caused by an acceptor ss mutation, has been shown to result in a significant increase in the enzyme activity. These promising results encourage further research into the therapeutic use of U1 snRNAs and chaperones to treat Sanfilippo syndrome type C patients.

## Availability of supporting data

Supporting data are included in the article and as additional files.

## Additional files

Additional file 1: Table S1. Oligonucleotide sequences for PCR amplifications. List of the oligonucleotides used in this work. Additional file 2: Figure S1. Electropherograms of the bands obtained in RT-PCR after transfection of the U1-sup4 in control and c.234 + 1G > A patient’s fibroblasts. (A) Wild type (control) sequence showing the identity of the normal fragment with exon 2 and 3. (B) Normal and aberrant sequence of the rescue band obtained with U1-sup4 transfection in patient’s fibroblasts. Additional file 3: Figure S2. PCR amplification to test the uptake of a modified U1 snRNA vector by patient fibroblasts. Agarose gel electrophoresis shows bands corresponding to the U1 vector after fibroblast transfection (by 2.5 μg and 3.5 μg of vector as indicated) and lower molecular weight bands which correspond to the *HGSNAT* exon 4 amplification, as a control. M: molecular weight marker; NT: non-treated cells; C-: negative control. Additional file 4: Table S2. Intron 15 donor ss scores using different predictors. Comparison of the ss scores for the normal site and the alternative site either in the presence or in the absence of the mutation.

## Abbreviations

Bp: Base pair
EET: Enzyme enhancement therapy
MPS III: Mucopolysaccharidoses type III
MPS IIIC: Mucopolyssacharidoses type IIIC or Sanfilippo syndrome type C
snRNAs: Small nuclear ribonucleic acids
ss: Splice site
WT: Wild type
