# Supplementary material for: Therapeutic strategies based on modified U1 snRNAs and chaperones for Sanfilippo C splicing mutations
Source: Orphanet J Rare Dis. 2014 Dec 10;9:180. doi: 10.1186/s13023-014-0180-y (PMC4279800; doi:10.1186/s13023-014-0180-y)
Supplement: Additional file 1: Table S1. — Oligonucleotide sequences for PCR amplifications. List of the oligonucleotides used in this work. [file 13023_2014_180_MOESM1_ESM.pdf]

Oligonucleotide	Sequence (5' to 3')
**RT-PCR fragments***	
HGSNAT-Exon 1F	GCAGCGGGCAGGCAAGGGCGG
HGSNAT-Exon 2F	ACATGCAGAGCTGAAGATGGA
HGSNAT-Exon 3R	GATAGATCCGTGCTGGGTG
HGSNAT-Exon 4F	AGCTGAACGACACCTTGGAA
HGSNAT-Exon 4R	CAATGATGACAGCAAGACCAA
HGSNAT-Exon 6F	TGCATTCCTTATTGGTCTTGC
HGSNAT-Exon 6R	ATCACCATCGAGAGGGTCTG
c.1542+4dupA F	ACCTGTGCCTGAACATTGTG
c.1542+4dupA R	CAGAAACCTTCGTCAGAGCA
HGSNAT-Exon 5F	CCTTTCTGAGGCTCTTGTTG
HGSNAT-Exon 12F	AGGAGCTGCCTTTCTCTTC
HGSNAT-Exon 13R	TGATCGTCTCCCAGCAGCA
HGSNAT-Exon 16R	ACCTTCGTCAGAGCAACAG
**DNA fragments***	
HGSNAT Exon 4 F	TTATTCTGCCTCCATGATATTAGC
HGSNAT Exon 4 R	CTACAGAAAGCGTCATGGACTGC
**Cloning fragments***	
Intron 1 F	GCAAAAGGAGACCTGTGTGTG
Intron 3 R	TCATCCCTGAGAACTGGCTTT
**Plasmid Vectors**	
SD6 F	TCTGAGTCACCTGGACAACC
SA2 R	ATCTCAGTGGTATTTGTGAGC
pGEMQ	ATCGAAATTAATACGACTCA
U1-QR	CTGGGAAAACCACCTTCGT
**Site-directed mutagenesis**	
**pSPL3 WT minigenes**	
Exon 2 F	GGAAATCTGAATGCTGTTATCACATATGTATCAGTTCACACTCAG
Exon 2 R	CTGAGTGTGAACTGATACATATGTGATAACAGCATTCAGATTTCC
Exon 6 F	CTGATCGCCTCATCAATTCTGTAAGTTATGAGATGCATAGTG
Exon 6 R	CACTATGCATCTCATAACTTACAGAATTGATGAGGCGATCAG
Exon 15 F	GGTGTTGTATTCTTGTAAGTAAGCAGCATTCCTCGC
Exon 15 R	GCGAGGAATGCTGCTTACTTACAAGAATACAACACC
**U1snRNA vectors**	
U1 suppressor 1 F	GATCTCATACTTATCTGGCAGGGGAGATAC
U1 suppressor 1 R	GTATCTCCCCTGCCAGATAAGTATGAGATC
U1 suppressor 2 F	GATCTCATACTTATGTGGCAGGGGAGATAC
U1 suppressor 2 R	GTATCTCCCCTGCCACATAAGTATGAGATC
U1 suppressor 3 F	CAAGATCTCATACATACGTGGCAGGGGAGATAC
U1 suppressor 3 R	GTATCTCCCCTGCCACGTATGTATGAGATCTTG
U1 suppressor 4 F	CAAGATCTCATACATATGTGGCAGGGGAG
U1 suppressor 4 R	CTCCCCTGCCACATATGTATGAGATCTTG
U1 suppressor 5 F	GATCTCATACTTATCTGGCAGGGGAGATAC
U1 suppressor 5 R	GTATCTCCCCTGCCAGATAAGTATGAGATC
U1 suppressor 6 F	GAGGCCCAAGATCTCTAACTTATAGAGCAGGGGAGATACCATG
U1 suppressor 6 R	CATGGTATCTCCCCTGCTCTATAAGTTAGAGATCTTGGGCCTC
U1 suppressor 7 F	AGGCCCAAGATCTCATACTTACAAGGCAGGGGAGATACC
U1 suppressor 7 R	GGTATCTCCCCTGCCTTGTAAGTATGAGATCTTGGGCCT
U1 suppressor 8 F	GCAGAGGCCCAAGATCTCAACTTTACCTGGCAGGGGAGATA
U1 suppressor 8 R	TATCTCCCCTGCCAGGTAAAGTTGAGATCTTGGGCCTCTGC
U1 suppressor 9 F	GTGTCGGGGCAGAGGCCCAAGATCTCAACTTTACAAGGCAGGGGAGATAC
U1 suppressor 9 R	GTATCTCCCCTGCCTTGTAAAGTTGAGATCTTGGGCCTCTGCCCCGACAC

* Primers were designed according to the sequence described in the ENSEMBL database (www.ensembl.org; ENSG00000165102). **F** – Forward; **R** – Reverse
