# Supplementary material for: Therapeutic strategies based on modified U1 snRNAs and chaperones for Sanfilippo C splicing mutations
Source: Orphanet J Rare Dis. 2014 Dec 10;9:180. doi: 10.1186/s13023-014-0180-y (PMC4279800; doi:10.1186/s13023-014-0180-y)
Supplement: Additional file 2: Figure S1. — Electropherograms of the bands obtained in RT-PCR after transfection of the U1-sup4 in control and c.234 + 1G > A patient’s fibroblasts. (A) Wild type (control) sequence showing the identity of the normal fragment with exon 2 and 3. (B) Normal and aberrant sequence of the rescue band obtained with U1-sup4 transfection in patient’s fibroblasts. [file 13023_2014_180_MOESM2_ESM.pdf]

A)

Wild-type sequence

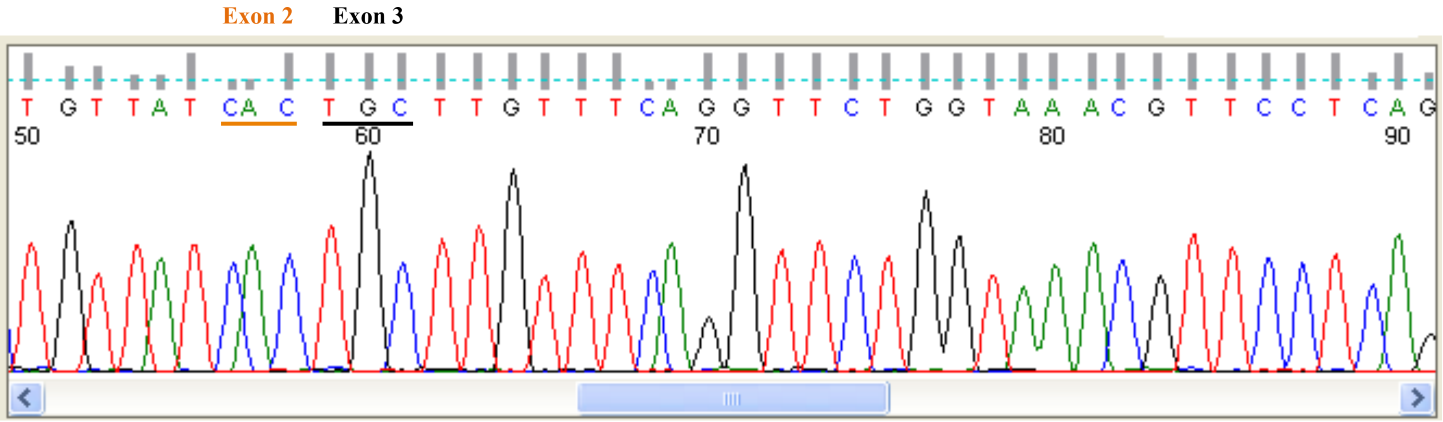

Normal exon 2 – exon 3 sequence ...TATCACTGCTTGTTTCAGGTTCTGGTAAACGTTCCTCAG...

B)

c.234+1G>A sequence after U1 sup 4 treatment

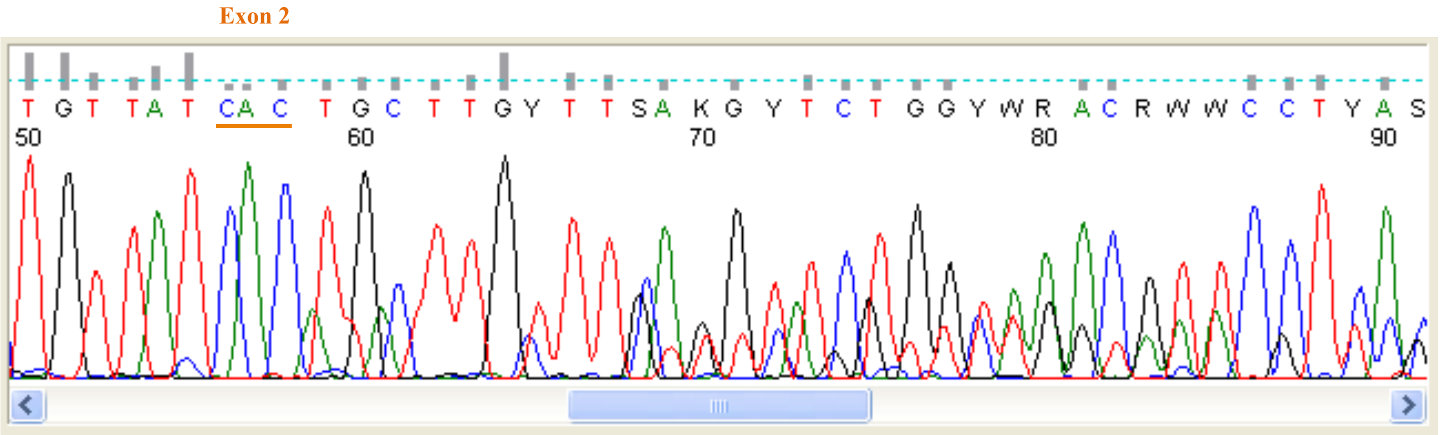

Two sequences:

Normal exon 2 – exon 3 sequence ...TATCACTGCTTGTTTCAGGTTCTGGTAAACGTTCCTCAG...

Aberrant exon 2 – exon 3 sequence ...TATCACatatTGCTTGTTTCAGGTTCTGGTAAACGTTCCTCAG...
