# Supplementary figures and images for: Therapeutic strategies based on modified U1 snRNAs and chaperones for Sanfilippo C splicing mutations
Source: Orphanet J Rare Dis. 2014 Dec 10;9:180. doi: 10.1186/s13023-014-0180-y (PMC4279800; doi:10.1186/s13023-014-0180-y)

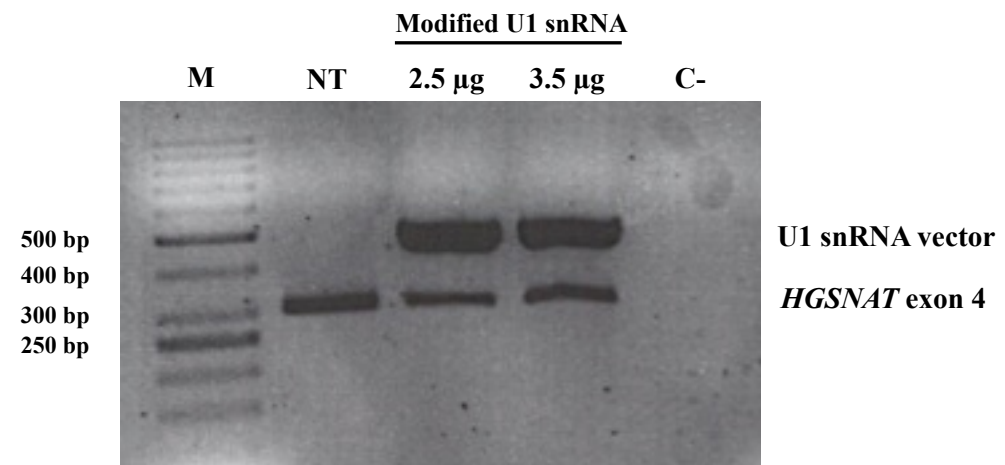

Supplement: Additional file 3: Figure S2. — PCR amplification to test the uptake of a modified U1 snRNA vector by patient fibroblasts. Agarose gel electrophoresis shows bands corresponding to the U1 vector after fibroblast transfection (by 2.5 μg and 3.5 μg of vector as indicated) and lower molecular weight bands which correspond to the HGSNAT exon 4 amplification, as a control. M: molecular weight marker; NT: non-treated cells; C-: negative control. [file 13023_2014_180_MOESM3_ESM.pdf]
