# Supplementary material for: Therapeutic strategies based on modified U1 snRNAs and chaperones for Sanfilippo C splicing mutations
Source: Orphanet J Rare Dis. 2014 Dec 10;9:180. doi: 10.1186/s13023-014-0180-y (PMC4279800; doi:10.1186/s13023-014-0180-y)
Supplement: Additional file 4: Table S2. — Intron 15 donor ss scores using different predictors. Comparison of the ss scores for the normal site and the alternative site either in the presence or in the absence of the mutation. [file 13023_2014_180_MOESM4_ESM.pdf]

Predictor	Normal	Alternative	Normal mutated	Alternative mutated
	CTT/gtaagt	taa/gtaagc	CTT/gtaaag	aaa/gtaagc
**Splice site score calculation (Max = 12.6)**[1]	6.9	6.2	2	7.6
**Analyzer splice tool (Max = 100)**[2]	78.94	78.17	61.92	81.99
**Splice site prediction by neuronal network (Max = 1) [29]**	0.95	0	0	0.97
**MaxEntScan (Max = 12) [30]**	8	5.66	-3	7.31
**Human splicing finder (Max = 100) [31]**	84.38	85.27	70.28	86.29

1. http://rulai.cshl.edu/new_alt_exon_db2/HTML/score.html
2. http://ibis.tau.ac.il/ssat/SpliceSiteFrame.htm
